# Effects of circulating tryptophan, kynurenine, kynurenic acid, IL-8, IFN-γ and IL-1β on neurocognition in clinically stable schizophrenia patients

**DOI:** 10.1192/j.eurpsy.2025.620

**Published:** 2025-08-26

**Authors:** M. Marković, T. Nikolić, M. Stašević, M. Stojković, M. Velimirović Bogosavljević, T. Stojković, S. Ristić, I. Stašević Karličić, S. Totić Poznanović, N. Petronijević

**Affiliations:** 1 Clinic for Psychiatry, University Clinical Centre of Serbia; 2 Institute for medical and clinical biochemistry; 3 Faculty of Medicine, University of Belgrade; 4 Clinic for Mental Disorders “Dr Laza Lazarević”, Belgrade; 5 Clinic for neurology, University Clinical Centre of Niš, Niš; 6 University of Belgrade, Belgrade; 7 Faculty of Medicine, University of Priština – Kosovska Mitrovica, Kosovska Mitrovica; 8 Ministry of Health, Belgrade, Serbia

## Abstract

**Introduction:**

Cognitive impairments represent core features in schizophrenia, impact the functional capacity of patients, and are highly predictive of poor functional outcomes. There is a huge unmet need for improvement of these impairments, and the development of new therapies is conditioned by understanding underlying pathophysiology that is not clear enough. Kynurenic acid (KYNA) is the major matabolite of kynurenine (KYN) pathway (KP) of tryptophan (TRP) degradation, which acts as the inhibitor of NMDA and α7-nicotinic receptors that are crucial for cognitive functioning. Excessive antagonism of these receptors by KYNA is hypothesized to contribute to cognitive deficits while data indicate that proinflammatory cytokyines activate this TRP catabolic cascade.

**Objectives:**

The objective of this study was to assess correlation between blood levels of TRP, KYNA, KYN, KYN/TRP ratio, IL-8, IFN-γ, IL-1β and cognitive functioning in clinically stable schizophrenia patients.

**Methods:**

We measured plasma concentrations of TRP, KYNA, KYN, IL-8, IFN-γ, IL-1β and conducted assessment of cognitive functioning in domains of verbal memory, working memory, attention and processing speed, motor speed, verbal fluency, and executive functions using Brief Assessment of Cognition in Schizophrenia Scale (BACS) in 64 clinically stable schizophrenia (SCZ) patients. Patients were matched by age, sex and body mass index and exclusion criteria included obesity class 2 or higher, any concomitant organic mental or neurological disorder, acute or chronic inflammatory disease, and use of immunomodulatory drugs or psychoactive substances.

**Results:**

A significant positive correlation was observed between BACS verbal fluency subtest score and kynurenine levels (p<0.05), along with a significant negative correlation with IL-β levels (p<0.05). There were no other significant correlations of blood levels of TRP, KYNA, KYN, KYN/TRP ratio, IL-8, and IFN-γ, and cognitive impairments in domains of verbal memory, working memory, attention and processing speed, motor speed, and executive functions.

**Conclusions:**

While the relationship between KP metabolites and cognitive functioning in schizophrenia in domains of verbal and working memory is more reported, specific correlation between KYN levels and verbal fluency observed in our study is less studied and understood. Furthermore, negative correlation between IL-1β levels and verbal memory performance is a valuable finding and can be further explored in the context of suggested detrimental role of IL-1β on hippocampal neurogenesis. Our findings should be interpreted cautiously and corroborated in larger studies with parallel measurement of preferal and central levels of analyzed parameters since there are data that suggest that peripheral concentrations of TRP, KYNA, KYN, IL-8, IFN-γ, IL-1β do not mirror those in the central nervous system.

**Disclosure of Interest:**

None Declared

